# DC-SIGN Mediates the Interaction Between Neutrophils and *Leishmania amazonensis*-Infected Dendritic Cells to Promote DC Maturation and Parasite Elimination

**DOI:** 10.3389/fimmu.2021.750648

**Published:** 2021-11-01

**Authors:** Rafael Tiburcio, Léon Dimitri Melo, Sara Nunes, Ana Luísa Augusto Barbosa, Elaine Carvalho de Oliveira, Martha Suarez, Valéria M. Borges, Natalia Tavares, Claudia Ida Brodskyn

**Affiliations:** ^1^ Instituto Gonçalo Moniz, Fundação Oswaldo Cruz, Salvador, Brazil; ^2^ Faculdade de Medicina da Bahia, Universidade Federal da Bahia, Salvador, Brazil; ^3^ Instituto de Investigação em Imunologia - Instituto de nacional de ciência e tecnologia (iii-INCT), São Paulo, Brazil

**Keywords:** leishmaniasis, polymorphonuclear cells, monocyte-derived dendritic cells, DC-SIGN, DC maturation markers

## Abstract

**Background:**

Leishmaniasis is a neglected arthropod-borne disease that affects millions of people worldwide. Successful *Leishmania* infections require the mitigation of immune cell functions leading to parasite survival and proliferation. A large body of evidence highlights the involvement of neutrophils (PMNs) and dendritic cells (DCs) in the establishment of immunological responses against these parasites. However, few studies, contemplate to what extent these cells interact synergistically to constrain *Leishmania* infection.

**Objective:**

We sought to investigate how PMNs and infected DCs interact in an *in vitro* model of *Leishmania amazonensis* infection.

**Material and Methods:**

Briefly, human PMNs and DCs were purified from the peripheral blood of healthy donors. Next, PMNs were activated with fibronectin and subsequently co-cultured with *L. amazonensis*-infected DCs.

**Results:**

We observed that *L. amazonensis*-infected DC exhibited lower rates of infection when co-cultivated with either resting or activated PMNs. Surprisingly, we found that the release of neutrophil enzymes was not involved in *Leishmania* killing. Next, we showed that the interaction between PMNs and infected-DCs was intermediated by DC-SIGN, further suggesting that parasite elimination occurs in a contact-dependent manner. Furthermore, we also observed that TNFα and ROS production was dependent on DC-SIGN-mediated contact, as well as parasite elimination is dependent on TNFα production in the co-culture. Finally, we observed that direct contact between PMNs and DCs are required to restore the expression of DC maturation molecules during *L. amazonensis* infection.

**Conclusion:**

Our findings suggest that the engagement of direct contact between PMNs and *L. amazonensis*-infected DC *via* DC-SIGN is required for the production of inflammatory mediators with subsequent parasite elimination and DC maturation.

## Introduction

Leishmaniasis is a neglected tropical disease caused by the infection of unicellular protozoans that belong to the genus *Leishmania*. According to the current World Health Organization estimates, approximately 12 million people are afflicted by leishmaniasis and 350 million are at risk of infection on a global scale (1–3). The major clinical subdivisions of leishmaniasis include chronic skin ulcer manifestations present in Tegumentary leishmaniasis (TL) or systemic inflammation of internal organs observed in Visceral Leishmaniasis (VL) (4–6). Of note, a broad array of factors such as parasite species, host genetic and health status influence disease outcomes (7–9).

During the first stages of *Leishmania* transmission, neutrophils, a category of polymorphonuclear cell (PMNs), are rapidly recruited to the site of infection, therefore being one of the first lineage of phagocytes to interact with these parasites (10). Of interest, PMNs comprise a versatile type of immune cells endowed with an antimicrobial arsenal which includes protease degranulation, augmented phagocytosis and induction of Neutrophil extracellular traps (NETs) (11). Nevertheless, a debate over the protective or detrimental role of PMNs in leishmaniasis remains unsettled. It is demonstrated that numerous aspects, including host genetic and pathogen biology, influence the outcome of pathogen-neutrophil interplay (10). Surprisingly, a mounting body of evidence suggest that apart from directly promoting the elimination of pathogens, PMNs are also able to influence the immunological functions of other leukocytes by promoting the migration of these cells to the site of infection, their activation, amplification of immunologic responses, and eventually pathogen elimination (11).

Numerous *in vitro* and *in vivo* studies demonstrate that successful *Leishmania* infections involve the impairment of Dendritic Cells (DCs) functions (12–15). DCs are professional antigen presentation cells not only in charge of bridging the innate and adaptive immune response but also of generating tolerance (16). Thus, targeting the immunological interactions between PMNs and DCs potentially offers new strategies to mitigate *Leishmania* infection.

In this study, we evaluated whether human PMNs influence the outcome of *Leishmania amazonensis* infection in DCs. Here, we showed that *L. amazonensis*- infected DCs co-cultivated with either activated or resting PMNs exhibited lower rates of infection and parasite load. Additionally, we observed that *L. amazonensis* elimination occurred in a direct contact-dependent manner mediated by DC-SIGN, a C-type lectin receptor found on DCs surface. Accordingly, we found that the interplay between these cells is important for both TNFα and ROS production. We observed that in settings of DC-SIGN mediated contact, ROS production was negatively correlated with parasite load. Conversely, ROS levels were positively correlated with TNFα amounts. Moreover, TNFα was directly involved in parasite elimination. Finally, we evaluated the impact of DC-neutrophil crosstalk in the expression of DC maturation molecules. Collectively, our findings suggest that the interaction between PMNs and DC play an important immunological role in constraining *L. amazonensis* infection and rescuing DC immune functions.

## Material and Methods

### Ethics Statement

This study was approved by the Institutional Review Board of Human Ethical Research Committee of Fundação Oswaldo Cruz-Bahia under the protocol number 1.213.510.

### Parasite Culture

*Leishmania amazonensis* MHOM/Br/00/BA125 strain was used for DCs infection. *L. amazonensis* promastigotes were maintained in Schneider culture medium (sigma^®^) supplemented with 10% fetal calf serum (sigma^®^), 2 mm L-glutamine, 100 μg/ml penicillin and 100 μg/ml streptomycin (sigma^®^) in an incubator at 24°c. During the culture period, promastigotes were counted in the Neubauer chamber with a 100x dilution in saline in order to measure parasite growth dynamics. In all experiments, stationary phase parasites were used which are enriched by infective stage of parasite, called metacyclic promastigotes.

### Isolation and Cultivation of Monocyte-Derived Dendritic Cells

Monocytes were obtained from the peripheral blood of healthy donors from the Bahia State Blood Center (HEMOBA). Blood was diluted in saline (1:1) and processed using the HISTOPAQUE^®^ 1077 separation gradient (Sigma Aldrich, St Louis, MO) for 30 minutes at 360xg at room temperature. After the centrifugation, a ring consisting of mononuclear cells was formed, collected and washed 3 times with saline at 200xg, 4°C. For differentiation into DCs, mononuclear cells were passed through magnetic separation columns for isolation of CD14^+^ cells (monocytes). These CD14^+^ cells were cultured for 7 days at a concentration of 1x10^6^/mL in complete RPMI medium (Gibco Invitrogen Corporation, Carlsbad, CA, USA) supplemented with 2mM L-glutamine, 100U/mL of penicillin and 100μX/mL streptomycin (complete medium) in the presence of IL-4 (100UI/mL) and GM-CSF (50ng/mL; both from PeproTech, Rocky Hill, NJ, USA) in 24- wells plates. Every 3 days of culture, 500 μL of medium containing growth factors were removed and replaced with the same amount of fresh medium.

### Dendritic Cell Infection

As previously described, stationary phase *L. amazonensis* promastigotes were used for *in vitro* infection of human dendritic cells. Infection assays were performed at the rate of 10 parasites for each dendritic cell (10: 1) in complete culture medium at 37°C, 5% CO_2_ for 24 hours. Subsequently, cells were centrifuged twice with saline solution to remove non-internalized parasites.

### Isolation and Culture of Human PMNs

PMNs were obtained from the peripheral blood of healthy donors from the Bahia State Blood Center (HEMOBA). Blood was processed using the Polymorphprep separation gradient as per manufacturer’s instructions (Axis-Shield Poc AS, Oslo, Norway). Briefly, the blood and gradient were centrifuged for 45 minutes at 300xg at room temperature. After centrifugation, PMNs were collected and washed three times with saline (0.9% NaCl) at 4°C for 10 minutes at 200xg. After washing, cells were counted in a Neubauer chamber with a final dilution of 100x in Trypan Blue stain. Cells were resuspended at a concentration of 5x10^5^/mL and cultured at 37°C in 5% CO2 in RPMI-1640 medium (Gibco Invitrogen Corporation, Carlsbad, CA, USA) supplemented with 2mM L-glutamine, 100U/mL of penicillin and 100μL/mL streptomycin (complete medium) (Gibco Invitrogen Corporation) for 96-well plate culture (Corning Incorporation, Costar, NY, USA).

### *In Vitro* Activation of PMNs With Extracellular Matrix Proteins

Neutrophil activation was induced by exposure of cells to culture plate surfaces coated with fibronectin extracellular matrix proteins. The activation protocol was adapted from Pinheiro et al. (17). Briefly, sterile 96-well culture plates (Corning Incorporation, Costar, NY, USA) were previously coated with 50μl of a 300μg/ml collagen solution (BD Pharmingem, San Diego, CA) and incubated for 12 hours at 4°C. To increase fibronectin binding to plate wells, 100μl of a 10μg/ml solution of human recombinant fibronectin (Sigma-Aldrich, St Louis, MO) was added to wells previously covered with collagen for 1 hour at 37°C. After sensitization with fibronectin, the wells were washed three times with saline and 2x10^6^ PMNs in RPMI medium were added to each well and incubated for 1 hour at 37°C and 5% CO_2_. After incubation with the matrix proteins, PMNs were collected and centrifuged with saline for 100xg 10 minutes at 4°C. Next, cells were counted and used in co-cultures with infected dendritic cells. Neutrophil treatment with enzyme inhibitors of human PMNs were plated at 5x10^5^ per well, and appropriate experimental groups were treated with myeloperoxidase (MPO) inhibitor (benzoic acid hydrazide analog, 0.1 mg/ml), TIMP (matrix metalloproteinase 9 [MMP-9] inhibitor, 30 ng/ml)or neutrophil elastase inhibitor (r & d, systems, Mineapollis, USA).

### Co-Culture Dendritic Cells - Activated PMNs

Infected dendritic cells were co-incubated with fibronectin-pre-activated PMNs in RPMI medium supplemented with 2 mM L-glutamine, 100 U/mL penicillin and 100 μg/mL streptomycin (Gibco, Invitrogen Corporation, Carlsbad, CA) for 4, 12 or 18 hours. In the interaction experiments, *L. amazonensis-* infected DCs and PMNs, a ratio of 5: 1 (PMNs: DCs) was used. After these periods, the wells were washed with saline, cells were submitted to cytospin centrifugation, fixed with ice-cold methanol for 10 minutes, and subsequently stained with hematoxylin-eosin (H&E). To determine the infection rate (number of DCs harboring at least one amastigote) and Parasite load (the number of amastigotes found in 100 cells), a total of 200 cells were counted per condition and in duplicate coverslips. For Transwell assays, *L. amazonensis* infected dendritic cells were accommodated in the lower compartment of Transwell plates (Sigma Aldrich, St Louis, MO) containing RPMI medium supplemented with 2 mM L-glutamine, 100 U/mL penicillin and 100 μg/mL streptomycin (Gibco, Invitrogen Corporation, Carlsbad, CA). In parallel, activated human PMNs were accommodated at the top of the Transwell plate. Importantly, DCs and PMNs were cultivated in transwell plates at the same ratio of 5: 1 (PMNs: DCs). The coculture was incubated at 37°C for 18 hours. Subsequently, DCs present in the lower portion of the plate were collected, washed with saline and the coverslips fixed with methanol for 20 minutes and stained with hematoxylin-eosin (H&E). Determination of infection rates and parasite load were performed as described above.

### Quantification of Soluble Mediators

After the determined interaction times between activated PMNs and *L. amazonensis* infected dendritic cells, the supernatants of such cocultures were collected to evaluate the production of cytokines and lipid mediators. Supernatants were tested for the presence of interleukins IL-6, IL-8, IL-12p70 and TNFα by ELISA (BD Biosciences,San Diego, CA) according to the manufacturer’s instructions. LTB_4_ and PGE2 lipid mediators were quantified by competition ELISA according to manufacturer’s instructions (CaymanChemicalCompany, MI, USA. Additionally, For MPO measurement, 50 µl fresh supernatant was added to a 96-well plate with 50 µl the developer solution ([5 ml] H2O2 30% + [10 ml] 1 mM citrate + 5 mg OPD) After 10–20 min, the reaction was stopped with 50 µl H_2_SO_4_ (8 N). MPO activity was measured by reading absorbance at 492 nm. For NE activity, 50 µl fresh supernatant was added in triplicate to ELISA plates, followed by 25 µl elastase reaction buffer (0.1 M HEPES, 0.5 M NaCl, 10% DMSO; all from Sigma-Aldrich; pH 7.5). Then, 0.2 mM elastase substrate I (MeOSuc-AAPV-pna) was added, and samples were incubated at 37°C for 3 d. NE activity was measured by reading absorbance at 410 nm.

### Antibody Neutralization of DC-Sign and TNFα

For DC-sign neutralizing assays, human DCs were initially infected with *L. amazonensis* for 24 hours in RPMI medium supplemented with 2 mM L-glutamine, 100 U/mL penicillin and 100 µg/mL streptomycin (Gibco, Invitrogen Corporation, Carlsbad, CA). Subsequently, DCs were centrifuged at 100xg for 10 minutes at 4°C to remove non-internalized promastigotes. Finally, activated human PMNs were added together with either 50ng/ml of the DC-SIGN neutralizing antibody (Abcam). For TNF-α neutralization, 10 µM of αTNF neutralizing antibody were used (Sigma Aldrich, St Louis, MO).

### Flow Cytometry Assay

Immunostaining of DCs for maturation molecules was performed by incubating cells with antiCD11c – PE-cy7, antiCD80- FITC, antiCD209- PE, antiHLA-DR – BV711, antiCD86 – PE-CY5, antiCD1a-AP antibodies (Bioscience), on ice, protected from light, for 30 minutes before to FACS analyses. Labelled DCs were acquired on BD LSR II flow cyometer (BD Biosciences). **Supplementary Table 1** summarizes all the details of flow cytometric antibodies used in this study. We used single color controls to further performed fluorochrome spillover compensation. **Supplementary Figure 3A** depicts gating strategy applied to analyze DCs. Briefly, DCs were initially gated based on FSC (forward scatter) and SSC (side scatter) channels. Next, we applied two sequential doublet exclusion gates to ensure the selection of single events (FSC-a *vs*. FSC-H and SSC-W *vs*. SSC-H). Then, Cells were gated based on CD11C expression. Median fluorescence intensity was calculated for each marker. For high dimensional analysis, 30000 DCs were downsampled to obtain subgroups with equal number of events. FlowSOM (Flow Self-organizing maps), an unsupervised clusters technique was employed to identify subpopulations of DCs with varied expression of maturation markers. Then, a t-Distributed Stochastic Neighbor Embedding (t-SNE) analysis were performed on gated DCs. ROS production was determined by flow cytometry (Dihydroethidium ROS Assay Kit, abcam).

### Statistical Analysis

Statistical analysis of the data was performed using the Prism software version 5.0 (GraphPad Software^®^). The data represent the medians obtained in each condition. Comparisons between groups were performed using Dunn’s post-test ANOVA test for analyzes with more than 3 groups and the Mann-Witney test (t-test) for the other comparisons. All experiments were performed with at least three repetitions and p-value <0.05 was considered statistically significant. The heatmaps shown in this study were generated with Pheatmap (pheatmap, RRID : SCR_016418) R package, version 1.0.12) in R language. Principal component analysis and lollipop plots presented in this study were constructed with factoextra and ggplot2 packages, respectively (18).

## Results

### *L. Amazonensis-*Infected DCS Co-Cultured With PMNs Display Reduced Infection Rates and Parasite Loads

**Figure 1:**
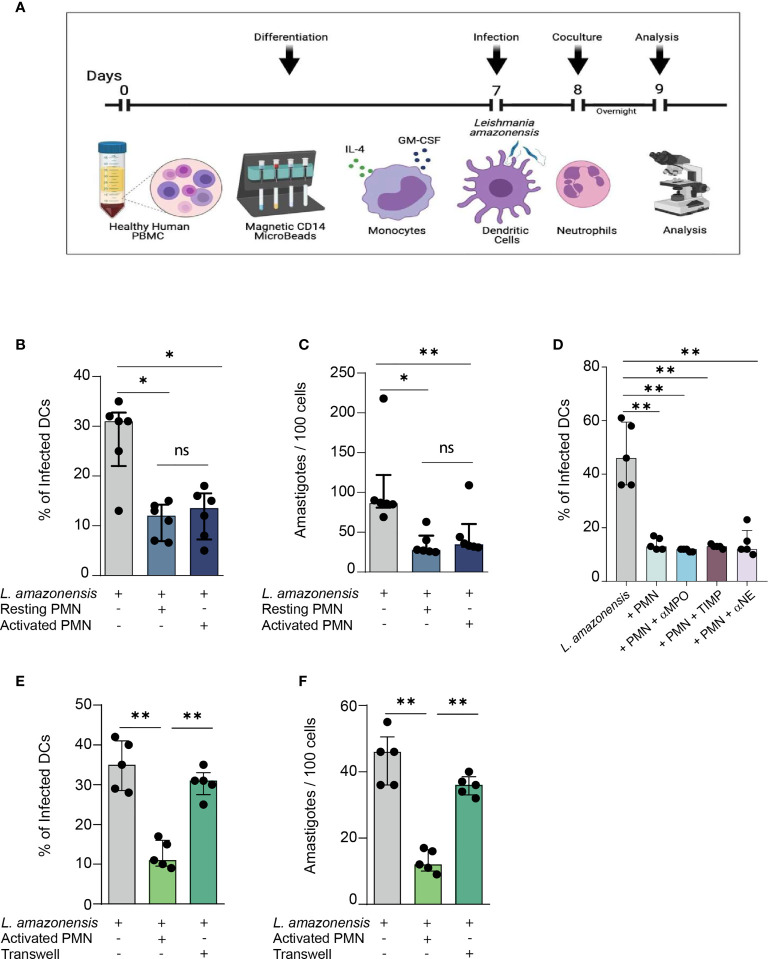
Relevance of direct contact between human PMNs and *L. amazonensis* – infected dendritic cells in constraining *in vitro* parasite infection. **(A)** Description of the experimental procedures. **(B)** Briefly, Monocyte-derived DCs were cultured with IL-4 and GM-CSF for 7 days. At the end of this period, fully-differentiated DCs were infected with metacyclic *L. amazonensis* promastigotes in the proportion of 10 parasites per cell. Subsequently, DCs were centrifuged to remove non-internalized parasite and incubated with fibronectin-activated neutrophils for 12 hours. Percentage of *L. amazonensis*-infected DCs co-cultured with either resting or activated PMNs. **(C)** Number of *L. amazonensis* amastigotes per 100 DCs, **(D)** Percentage of infected DCs in cultures treated with inhibitors of neutrophil enzymes. **(E, F)** Infection rate and parasite load in transwell assays. n = 6. Kruskal-Wallis test with Dunn post-test. (ns = non-significant). Each dot represents a donor n = 5, **p < 0.01, *p < 0.05.

Initially, we assessed whether human PMNs were able to influence the outcome of *L. amazonensis* infection in DCs, and if such infection constrain was dependent on the state of neutrophil activation. Briefly, DCs were infected with *L. amazonensis* for 24 hours and subsequently cultivated with either fibronectin-activated or resting PMNs for 12 hours as depicted in our experimental design scheme (**Figure 1A**). We verified that DCs co-cultivated with resting or activated PMNs exhibited diminished rates of infection as well as decreased numbers of *Leishmania* amastigotes found per 100 DCs (**Figures 1B, C**). We did not find statistically significant differences between the groups of resting or activated PMNs co-cultivated with DCs, suggesting that induction of parasite elimination is independent of the neutrophil activation state. Therefore, to mimic the biological context in which PMNs and DCs interact during *Leishmania* infection, we decided to only use activated PMNs for the subsequent experiments.

Next, we sought to explore the mechanisms through which PMNs promote parasite elimination in infected DCs. Initially, we measured the levels of neutrophil enzymes produced in the co-culture of *Leishmania*-infected DCs and either resting or activated PMNs. We detected higher levels of both MPO and neutrophil elastase (NE) in the co-culture of DCs and activated PMNs (**Supplementary Figures 1A, B**). Surprisingly, the pharmacological inhibition of individual neutrophil enzymes did not alter the infection rate (**Figure 1D**), suggesting that contact-independent mechanisms are not responsible for the parasite elimination we observed in neutrophil-DC interaction. Thus, we aimed to investigate to what extent the constrain of *Leishmania* infection in DCs was mediated by contact-dependent mechanisms. Accordingly, to avoid physical contact between the cells, we co-cultured DCs and PMNs in different compartments of transwell plates. We observed that DCs co-cultured in such plates exhibited higher rates of infection and parasite load when contrasted to the groups in which PMNs and DCs were free to establish direct contact (**Figures 1E, F**). Thus, our findings suggest that parasite elimination is dependent on the physical contact mechanism.

### DC-Sign Mediates Direct Contact Between PMNs and DCS

**Figure 2:**
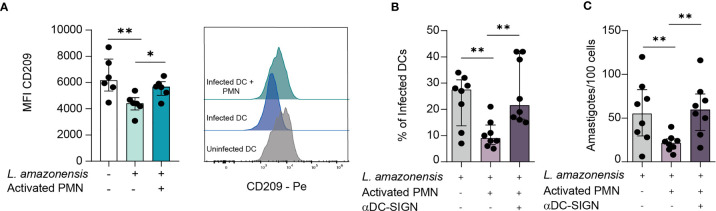
DC-SIGN-mediated contact between DCs and PMNs is important to mitigate *L. amazonensis* infection. DCs were infected for 24 hours with *L. amazonensis* and subsequently incubated with activated neutrophils for 12 hours. **(A)** Median fluorescence intensity of CD209 (DC-SIGN) measured by FACS. Right panel depict the histogram of CD209 MFI, n = 6. **(B, C)** Infection rates and parasite load of infected DCs in cultures treated with DC-SIGN inhibitor. n = 8. Kruskal-Wallis test with Dunn post-test. n = 8. Each dot represents a donor. **p < 0.01, *p < 0.05.

Dendritic Cell-Specific Intercellular adhesion molecule-3-Grabbing Non-integrin (DC-SIGN) is a major orchestrator in DCs-Neutrophil crosstalk (19). A wealth of evidence points that DC-SIGN, a C-type lectin present on DCs surface, recognizes Lewis X glycan moieties on PMN receptors, thus mediating the establishment of immunological synapses between these two types of cells. It is well established that the DC-PMN cross-talk culminates in reciprocal modulation of their immune functions (by influencing processes such as Cell activation, cytokine production, and resistance to apoptosis) (19). Therefore, we sought to elucidate whether DC-SIGN-mediated contact plays a role in *Leishmania* elimination. Initially, we measured the surface expression of DC-SIGN in infected DCs. Of note, we observed a significant reduction of DC-SIGN levels in *L. amazonensis*-infected DCs, and that co-culture with PMNs was able to rescue this receptor expression (**Figure 2A**). Additionally, we found elevated rates of infectivity and parasite burden upon *in vitro* treatment with DC-SIGN neutralizing antibody, further suggesting that such receptor is a paramount mediator of the physical contact-dependent parasite elimination (**Figures 2B, C**).

### DC-Sign Mediated Contact Regulates the Production of Soluble Mediators and Reactive Oxygen Species

**Figure 3:**
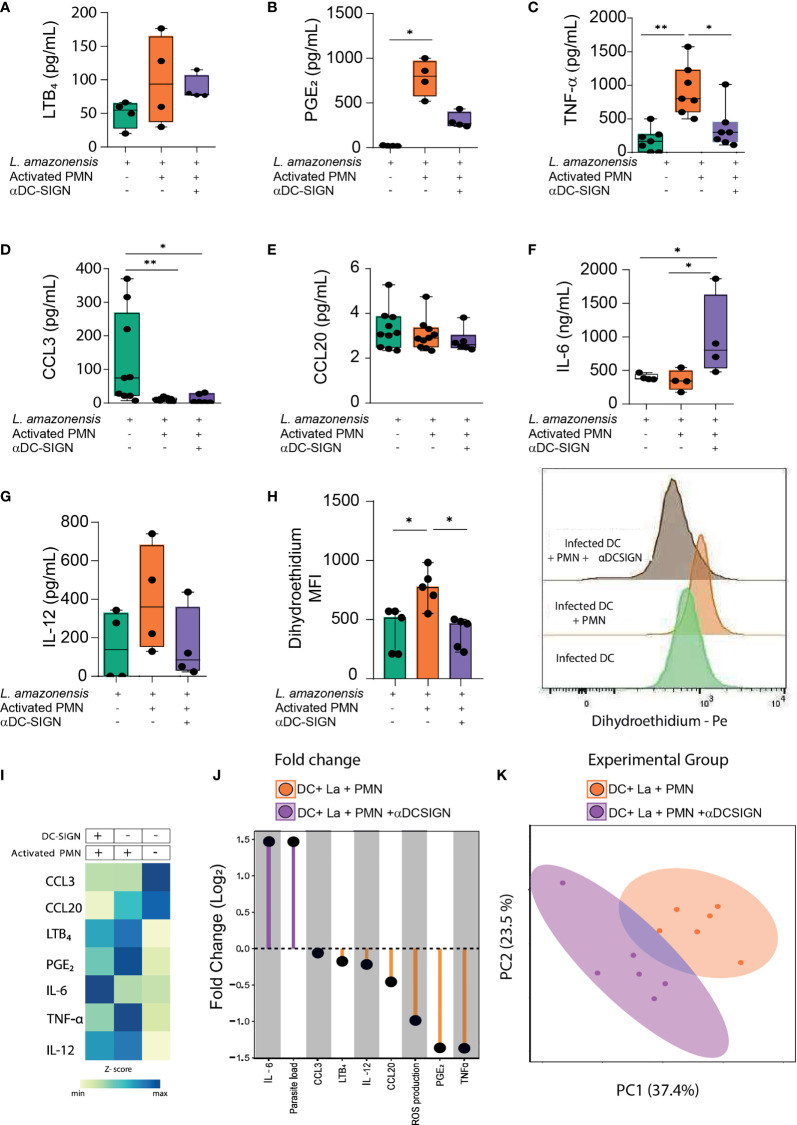
Soluble mediators and ROS production depends on interaction *via* DC-SIGN. **(A–G)** Briefly, cell culture supernatants from different experimental conditions were collected and submitted to ELISA in order to quantify the production of LTB_4_, PGE_2_, TNFα, CCL3, CCL20, IL-6, and IL-12. **(H)** Reactive oxygen species was measured in gated Dendritic cells by dihydroethidium (DHE) MFI measured by FACS. Right panel depicts the histogram of DHE MFI. **(I)** Heatmap summarizes the median values of soluble mediators present in cells cultures supernatants. **(J)** Lollipop chart compares the log2-fold change of soluble mediators, parasite load, and ROS production in different experimental conditions (DC-SIGN inhibited Vs not inhibited). **(K)** Principal component analysis of soluble mediators and ROS production levels was conducted. n = 6. Kruskal-Wallis test with Dunn post-test. Each dot represents a donor, **p < 0.01, *p < 0.05.

Next, we wished to evaluate the contribution of DC-SIGN-mediated crosstalk between DCs and PMNs in the production of soluble mediators (namely, chemokines, lipid mediators, and cytokines). Concerning eicosanoid production, we observed increased secretion of both LTB_4_ and PGE_2_ when DCs were cultured with PMNs, but we could only detect statistically significant enhanced amounts of the latter lipid mediator (**Figures 3A, B**). Additionally, upon DC-SIGN neutralization, PGE_2_ levels were only marginally increased when contrasted to monocultures of infected DCs (**Figure 3B**). We also detected enhanced levels of TNF-α in DC-Neutrophil cultures, with decreased production upon DC-SIGN inhibition (**Figure 3C**). Interestingly, the amounts of CCL3 were substantially reduced in the presence of human PMNs, while no significant difference in CCL20 production was observerd among the experimental groups (**Figures 3D, E**). Notably, we observed that DC-SIGN neutralization was associated with augmented production of IL-6 when compared to other experimental conditions (**Figure 3F**). Additionally, the addition of PMNs to infected-DCs culture did not result in elevation of IL-12 levels (**Figure 3G**). Collectively, our observations suggest that the establishment of direct contact between DCs and PMNs orchestrates the production of several soluble mediators in the settings of *L. amazonensis* infection. Subsequently, we investigated whether such cell-cell engagement *via* DC-SIGN is necessary for the fine-tuning induction of reactive oxygen species (ROS). Of note, we observed that the addition of activated PMNs to infected DCs cultures promoted enhanced levels of ROS production and DC-SIGN neutralization had a reverse effect (**Figure 3H**). Additionally, we applied a heatmap with the median values of the soluble mediators as an approach to identify patterns among the experimental conditions (**Figure 3I**). To better understand to what magnitude the contact *via* DC-SIGN regulates soluble mediator production, we devised an approach comparing the fold changes in cultures where DC-SIGN was inhibited *versus* not inhibited. Interestingly, we found a 1.5-fold higher increase of both IL-6 levels and the number of amastigotes in experimental conditions where DC-SIGN was neutralized. Conversely, we detected a 1.5-fold decrease in PGE_2_ and TNFα production in the aforementioned conditions (**Figure 3J**). Additionally, our principal component analysis showed little overlap between these groups. Importantly, we found that TNFα levels positively correlate with ROS production, while we could detect negative correlations between ROS levels and the amounts of amastigotes in infected DCs (**Supplementary Figure 2**). Therefore, these observations suggest that the constrain of *L. amazonensis* infection promoted by the interaction with PMNs is dependent on the TNFα and may be associated with ROS activity. Collectively, our data indicate that DC-SIGN-mediated contact can substantially influence both production of soluble molecules and ROS induction with eventual impact in *L. amazonensis* elimination in DCs (**Figure 3K**).

### TNFα Produced in PMNs AND DCs Co-Culture Is Required for Parasite Killing

**Figure 4:**
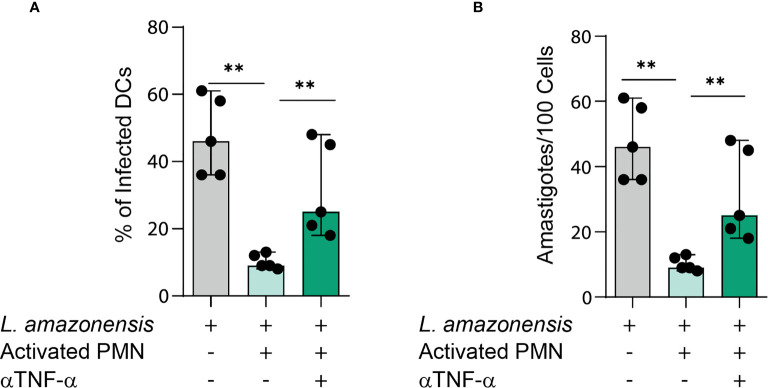
TNFα is required for parasite elimination in PMNs-DCs cultures. **(A, B)** Infection rates and parasite load of infected DCs in cultures treated with TNFα neutralizing antibody. Kruskal-Wallis test with Dunn post-test. Each dot represents a donor, n = 5, **p < 0.01, *p < 0.05.

TNFα is an important pro-inflammatory cytokine involved in cell activation and elimination of pathogens (20). We next ascertained whether TNFα contributes to *L. amazonensis* elimination in the studied co-culture model. Thus, we treated cell cultures with TNFα neutralizing antibody and measured the percentages of infected DCs as well as the number of amastigotes. Of note, we observed that the treatment with TNFα neutralizing antibody resulted in enhanced infection rates and parasite load when contrasted to the PMN- infected DC cultures not treated with such antibody (**Figures 4A, B**). These findings underscore the importance of TNFα in the control of *L. amazonensis* infection in the setting of DCs-neutrophil interaction.

### PMNs Influence the Maturation of *L. Amazonensis* -Infected DCS VIA DC-Sign

**Figure 5:**
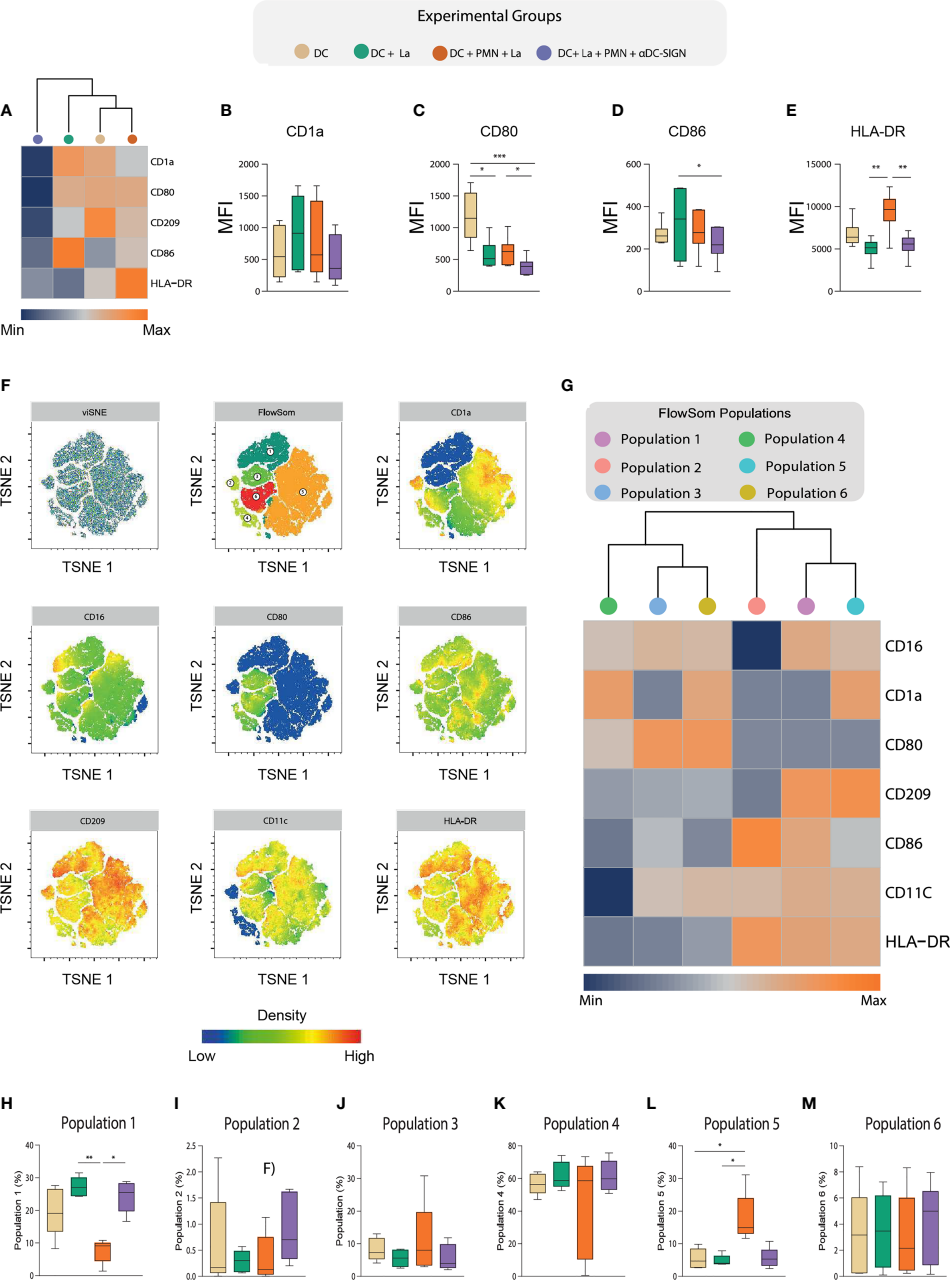
Deep characterization of DC maturation immuneprofile *via* High dimensional flow cytometry. **(A)** Hierarchical cluster analysis with heatmap depicts the expression of HLA-DR,CD80,CD86,CD1a, CD16, and CD209 in the experimental groups. **(B–E)** Boxplot indicates the MFI of the aforementioned molecules in our experimental groups. **(F)** t-distribute stochastic neighbor embedding (tSNE) and FlowSOM clusterization considering the expression of HLA-DR,CD80,CD86,CD1a, CD16, and CD209 after concatenation of all experimental groups. **(G)** A hierarchical cluster analysis with heatmap depict the expression of the aforementioned markers in the populations identified by FlowSom clustering. **(H–M)** Frequencies of each FlowSOM identified population across different experimental conditions. Each dot represents a donor, n = 5. Kruskal-Wallis test with Dunn post-test, **p < 0.01, *p < 0.05.

Presenting antigens to adaptive immune cells is a critical immunobiological function of DCs (21). Accordingly, several species of *Leishmania* developed mechanisms to mitigate expression of molecules involved in this process, thus enhancing the chances of parasite survival and proliferation (22). Along these lines, we employed a flow cytometry approach to investigate to what degree the interaction between DCs and PMNs *via* DC-SIGN impacts the process of DC maturation. To analyze the maturation profiles, we took into consideration the expression of HLA-DR and costimulatory molecules (CD1a, CD80, and CD86), as well as CD209 (DC-SIGN) (**Figure 5A**). Of note, we found that *L. amazonensis* infection is associated with increased numbers of CD1a^+^ DCs and that the presence of PMNs reduced the size of these cell population in a DC-SIGN independent manner (**Figure 5B** and **Supplementary Figure 3B**). For the other markers, we could not observe significant variation in percentage of cells expressing such molecules (**Supplementary Figure 3B**).

Nevertheless, by analyzing the bulky population of DCs, we found that *L. amazonensis* infection decreased CD80 surface density (measured as the median value of fluorescence intensity), and that PMNs could partially upregulate this co-stimulatory molecule depending on DC-SIGN -promoted contact (**Figure 5C**). Strikingly, we noticed a CD86 upregulation in infected DCs (**Figure 5D**). Most interestingly, we observed that PMNs are able to upregulate the expression of HLA-DR in a DC-SIGN dependent fashion (**Figure 5E**). Collectively, our data suggest that the engagement of DCs and neutrophil *via* DC-SIGN is associated with partial reconstitution of maturation-associated molecules in settings of *L. amazonensis* infection.

As environmental cues largely influence the ability of DCs to present antigens, we hypothesized that co-culture with PMNs would give rise to DC populations with varying levels of such molecules expression in the context of *L. amazonensis* infection. Thus, we applied a t-distribute stochastic neighbor embedding (tSNE) analysis to depict co-stimulatory molecule expression profiles found in the overall experimental groups (**Figure 5F**) and among the different conditions (**Supplementary Figure 4**). Notably, our FlowSom clustering was able to detect 6 populations with distinct maturation profiles (**Figure 5F**). We then conduct a heatmap with hierarchical cluster analysis to better dissect these population in regard to marker expression and distribution across experimental conditions (**Figures 5G**). Interestingly, we observed significant enrichment of two population clusters (populations 1 and 5) in the co-culture of DCs and PMNs (**Figures 5H–M**). The frequency of DCs referred as population 1 (CD16^high^, CD1a ^dim^, CD80 ^dim^, CD209 ^high^, CD86 ^int^, CD11c ^int^, and HLA-DR ^high^) was increased in *L. amazonensis*-infected DCs and PMNs were able to promote its frequency reduction in a DC-SIGN dependent manner(**Figure 5H**). Conversely, we noticed that population 5 (CD16 ^int^, CD1a ^high^, CD80 ^dim^, CD209 ^high^, CD86 ^dim^, CD11c ^int^, and HLA-DR ^high^) was increased in the condition co-cultured with PMNs (**Figure 5M**).

**Figure 6:**
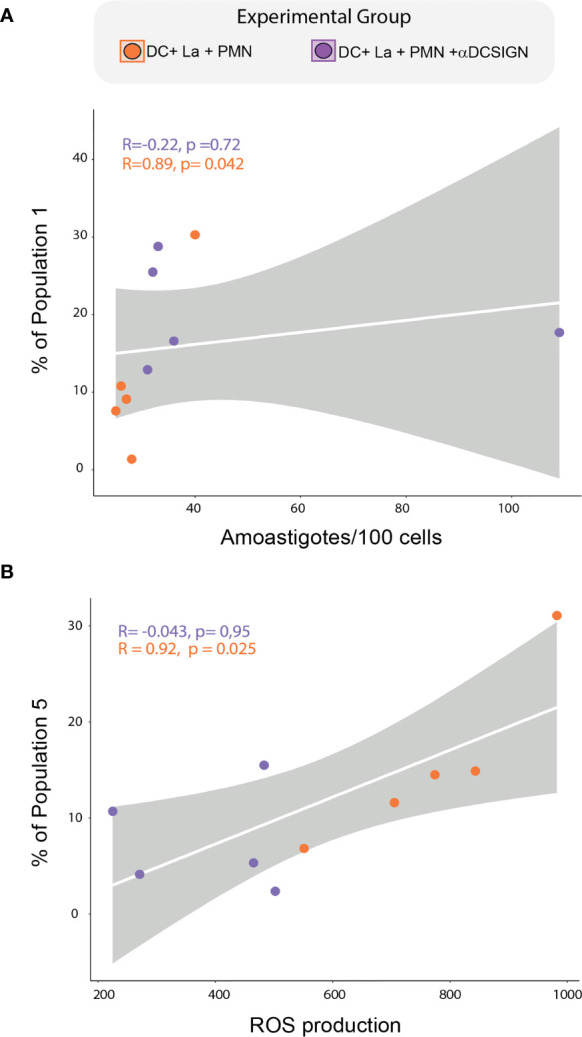
Correlation between DCs subpopulations, Parasite load, and ROS production. **(A)** Spearman correlation between FlowSOM-identified population 1 and parasite load. **(B)** correlation between the frequency of FlowSOM-identified population 5 and ROS production. Correlations were tested by a two-tailed non-parametric Spearman rank test. Each dot represents a donor.

Additionally, our spearman correlation analysis revealed a positive correlation between the frequencies of FlowSom population 1 and parasite load, while population 5 was positively correlated with ROS production in infected DC cultured with PMNs (**Figures 6A, B**). Of note, we this was not observed in aDC-SIGN treated cell cultures. We were not able to find significant correlation between the frequency these populations and the level of soluble molecules produced during neutrophil and DC co-culture. Collectively, our data suggest that DC-SIGN inhibition in co-cultures of PMNS and infected DCs can influence the DC maturation landscape, giving rise to populations associated either with ROS production or parasite survival.

## Discussion

Here we report the effects of the interaction between human PMNs and *L.amazonensis* - infected DCs. Our data indicate that PMNs contribute to the control of *L. amazonensis* infection in DCs as evidenced by decreased infection rates and parasite loads in cocultures. Of note, PMNs comprise the first line of defense against a wide array of pathogens and acquire an activated state upon encounter with molecular patterns associated with pathogens or danger (PAMPS and DAMPS, respectively) (23). Bearing in mind that activated PMNs are endowed with effective antimicrobial functions and capable of modulating other immune cells functions, we analyzed the relevance of PMN activation in the crosstalk with infected DCs. Interestingly, the control exerted by PMNs was independent of their activation state. It is well established that interactions between PMNs and other leukocytes is relevant for the outcome of *Leishmania* infections. Previously, our group showed that interactions between PMNs and *L. amazonensis* -infected macrophages can be either detrimental *via* TGF-β1 and PGE_2_ or contribute to parasite elimination depending on TNF-α activity. In this case, PMNs viability dictated the outcome of *Leishmania* infection in macrophages (24).

We sought to dissect the mechanisms through which PMNs could induce *L. amazonensis* elimination in DCs. Thus, we devised an *in vitro* assay of pharmacological inhibition of several neutrophil enzymes. Strikingly, our data demonstrates that the increase in parasite elimination probably occurs independently of neutrophil degranulation. A previous report of our group showed that granules released from PMNs activate *L. amazonensis*-infected macrophages *via* Toll-like receptor signaling resulting in augmented production of inflammatory molecules and reduced parasite burden (25). In our study, however, it was shown that leishmanicidal activity in DCs induced by PMNs were largely dependent on direct cell-cell contact. Importantly, further experimental approaches should target PMN-mediated decrease in parasite load in DCs to reveal whether PMNs promote a bona fide *L. amazonensis* elimination or an amastigote growth arrest after coculture.

This study also highlights that the cross-talk between PMNs and infected DCs is mediated by DC-SIGN. Several reports underscore the multifaceted role of DC-SIGN in immune functions of DCs, including recognition and binding to carbohydrate motifs found on several pathogens, other leukocytes, and even self-antigens (26–30). Our data demonstrate that *L. amazonensis* infection is associated with a significant reduction in DC-SIGN expression, which is rescued in the presence of activated PMNs. We hypothesize that DC-SIGN surface expression is reduced in the infected DCs because this receptor is trapped in endosomes following *Leishmania* internalization. In cocultures, DC-SIGN upregulation may occur *via* receptor recycling that ensues PMN- induced parasite elimination. It is important to note that further experiments are required to confirm these hypothesis. Additionally, our data demonstrate that neutralizing DC-SIGN results in increased parasite burden in DCs, thus pointing the importance of DC-SIGN -mediated communication between PMNS and DCs in constrain of an *in vitro* model of *Leishmania* infection. Importantly, It has been demonstrated that macrophage -1 antigen (MAC-1), an integrin present on the surface of PMNs, is an important ligand for DC-SIGN (31). A pioneering study conducted by Gisbergen et al. demonstrated a role for MAC-1/DC-SIGN -mediated cell adhesion between immature DCs and activated PMNs with substantial effects in tailoring adaptive immunity (31).

A paramount function of DCs is the production of specific cytokines that concatenate innate and adaptive branches of immunity (32). In light of this, we investigated the inflammatory molecules found in coculture supernatant. We show that PGE_2_ production occurs in a manner dependent of DC-SIGN mediated contact. More importantly, we also observed a similar pattern for TNF-α secretion. Tavares et al. reported a role for TNF-α in *L. amazonensis* elimination in the context of neutrophil and macrophages cross-talk (24). Along this line, others demonstrated that macrophages prompt neutrophil apoptosis *via* membrane TNF-α in *L. major* infected mice (33). Additionally, we show that *L. amazonensis* elimination in DCs depends on TNF-α. These works corroborate our findings that TNF-α derived from the interactions between PMNs and mononuclear myeloid cells influence substantially the outcomes of *Leishmania* infections. Unexpectedly, we found that IL-6 production is enhanced upon DC-SIGN neutralization. Other reports that DC-SIGN binding to SALP15, an immunosuppressive molecules found in tick saliva, results in decrease of IL-6 and TNF-α mRNA stability in *via* Raf-1/mitogen-activated protein kinase kinase (MEKK) axis (34). In light of the above findings, we hypothesize DC-SIGN inhibition itself results in augmented levels of IL-6 in the context of *L. amazonensis* infection. However, further studies are necessary to investigate whether DC-SIGN binding to *Leishmania* spp components prompts signaling pathways that result in decreased IL-6 production.

It is worth noting that ROS production was dependent on DC-SIGN-mediated contact. We also observed that ROS levels and the parasite burden were negatively correlated. Conversely, ROS production positively correlated with TNF- α levels. A work conducted by Carneiro et al. demonstrated that ROS inhibition in *L. braziliensis*-infected monocytes from Cutaneous Leishmaniasis patients mitigated parasite elimination (35) Nevertheless, Roma and colleagues showed that ROS is not required for *L. amazonensis* constrain in peritonial macrophages from C57BL/6 mice (36).

By the means of physical interactions, PMNs modulate substantially DC maturation. A seminal study conducted by Van Gisbergen and colleagues demonstrate a role for DC-SIGN in PMN-promoted DC maturation (32). Here we observed a PMN-induced signature in DC expression of co-stimulatory molecules which is dependent of cell-cell contact. Particularly, physical contact allowed both HLA-DR and CD80 upregulation, which counteracts the effects of *Leishmania* infection on these molecules. Notably, Figueiredo et al. demonstrated that *L. amazonensis* triggers MHC II and CD86 downregulation in a CD39/CD73/adenosine axis dependent manner (37).

We also employed a high dimensional flow cytometry approach to dissect the DC maturation landscape in cocultures. The chosen flow cytometry gating strategy allowed for filtering out PMNs, while observing maturation molecules expressed only in DC subpopulations. Importantly, we identified a CD209^high^, CD1a^high^, HLA-DR^high^ subpopulation whose frequencies are enhanced upon physical PMN-DCs interactions. Additionally, this subpopulation was positively correlated with ROS production. On the other hand, we noticed decrease in the percentage of a subpopulation CD209^high^, CD1a^dim^, HLA-DR^high^ that was dependent on DC-SIGN mediated contact. We found that this latter population was positively correlated with the number of amastigotes. Altogether, our data demonstrate that PMNs induce a partial restoration of maturation-associated molecules in *L. amazonensis* infected DC. Additional experiments are needed to assess whether the PMN-induced maturation occurs in infected or bystander DCs.

A major limitation of this study is that we develop our conceptual framework around an *in vitro* model of *Leishmania* infection and Cell interactions. Additionally, the events observed may be dependent on the strain of *Leishmania* chosen. Importantly, the effects of PMN-induced maturation of DCs may also impact T lymphocytes polarization. Therefore, other *in vivo* and *in vitro* studies are warranted to be conducted to unravel these queries.

**Figure 7:**
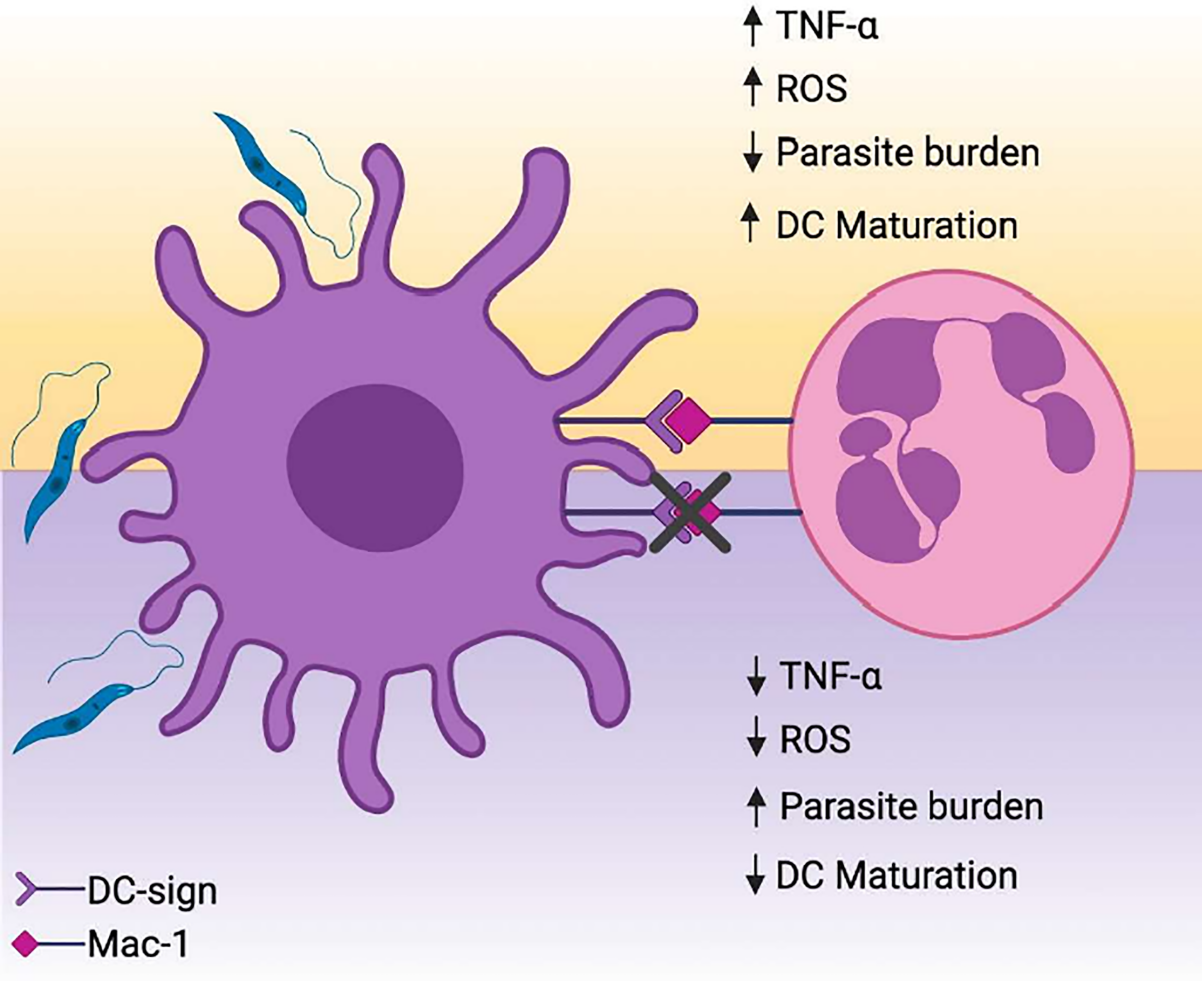
PMNs promote parasite elimination and partial upregulation of maturation molecules in *L. amazonensis*-infected DCs. **(A)** *L. amazonensis* infection results in substantial mitigation of DCs immunobiological functions, hampering the differentiation of these cells into a mature state and eventual ablation of antigen presentation. The interaction between activated PMNs and infected DCs *via* DC-SIGN drives parasite elimination in a manner dependent of ROS/TNFα axis. Concomitantly, DC-SIGN mediated crosstalk is associated with upregulation of maturation-related and co-stimulatory molecules on DCs. Created with Biorender.com.

Put together, the results reported in our study have identified the contributions of DC-SIGN mediated contact with DCs in constraining *L. amazonensis* infection. This communication resulted in decreased parasite burden, augmented TNF-α production and enhanced expression of DC maturation markers (**Figure 7**). Collectively, the findings presented here contribute to an enhanced understanding of nuances in PMNs and DCs biology in surrounding Leishmanasis.

## Data Availability Statement

The raw data supporting the conclusions of this article will be made available by the authors, without undue reservation.

## Ethics Statement

The studies involving human participants were reviewed and approved by Committee of Research Ethics-Instituto Gonçalo Moniz. The patients/participants provided their written informed consent to participate in this study.

## Author Contributions

RT conceived and carried out the experiments and wrote the manuscript. LM, EO, AB, and MS carried out thee experiments. VMB and NT conceived the idea and design the experiments. CB: Conceived the idea, supervise and analyse the results, wrote the manuscript. All authors contributed to the article and approved the submitted version.

## Funding

This work was supported by the National Council of Research (CNPq–Universal) grants 476926/2011-4 for CB and FAPESB APP0108/2016 for VMB. VMB and CB are senior investigators from CNPq. The funders had no role in study design, data collection and analysis, decision to publish, or preparation of the manuscript.

## Conflict of Interest

The authors declare that the research was conducted in the absence of any commercial or financial relationships that could be construed as a potential conflict of interest.

## Publisher’s Note

All claims expressed in this article are solely those of the authors and do not necessarily represent those of their affiliated organizations, or those of the publisher, the editors and the reviewers. Any product that may be evaluated in this article, or claim that may be made by its manufacturer, is not guaranteed or endorsed by the publisher.

## Supplementary Material

Supplementary Figure 1 Assessment of Neutrophil enzymes activity in PMN-infected DCs cocultures. The enzymatic activity of MPO **(A)** and Neutrophil elastase **(B)** were measured in coculture supernantants. Kruskal-Wallis test with Dunn post-test. *p < 0,05.

Supplementary Figure 2 Spearman correlations between TNFα and ROS production. Briefly, monocyte-derived DCs were cultured with IL-4 and GM-CSF for 7 days. At the end of this period, fully-differentiated DCs were infected with metacyclic *L. amazonensis* promastigotes in the proportion of 10 parasites per cell. Subsequently, DCs were centrifuged to remove non-internalized parasite and incubated with fibronectin-activated neutrophils for 12 hours. Supernatant was collected for assessment of TNFα production. ROS production was determined by flow cytometry. *p < 0,05. Correlations were tested by a two-tailed non-parametric Spearman rank test.

Supplementary Figure 3 Flow cytometry analysis of maturation -related molecules in DCs. **(A)** Gating strategy for DCs identification and **(B)** frequency of CD1a, CD80, CD86, and HLA-DR expression. Kruskal-Wallis test with Dunn post-test. *p < 0,05.

Supplementary Figure 4 High dimensional flow cytometry characterization of DC subpopulations. **(A)** t-distribute stochastic neighbor embedding (tSNE) and FlowSOM clusterization considering the expression of HLA-DR,CD80, CD86, CD1a, CD16, and CD209 across experimental conditions.

## References

[1] Alvar J Vélez ID Bern C Herrero M Desjeux P Cano J. Leishmaniasis Worldwide and Global Estimates of Its Incidence. PLoS One (2012) 7(5):e35671. doi: 10.1371/journal.pone.0035671 22693548 PMC3365071

[2] Desjeux P. Leishmaniasis: Current Situation and New Perspectives. Comp Immunology Microbiol Infect Dis (2004) 27(5):305–18. doi: 10.1016/j.cimid.2004.03.004 15225981

[3] World Health Organization. Control of the Leishmaniases. World Health Organ Tech Rep Ser (2010) 949. 21485694

[4] Amato VS Tuon FF Bacha HA Neto VA Nicodemo AC. Mucosal Leishmaniasis. Current Scenario and Prospects for Treatment. Acta Tropica (2008) 105(1):1–9. doi: 10.1016/j.actatropica.2007.08.003 17884002

[5] Scott P Novais FO. Cutaneous Leishmaniasis: Immune Responses in Protection and Pathogenesis. Nat Rev Immunol (2016) 16(9):581–92. doi: 10.1038/nri.2016.72 27424773

[6] de Oliveira CI Brodskyn CI. The Immunobiology of Leishmania Braziliensis Infection. Front Immunol (2012) 3:145. doi: 10.3389/fimmu.2012.00145 22701117 PMC3370302

[7] Kaye P Scott P. Leishmaniasis: Complexity at the Host-Pathogen Interface. Nat Rev Microbiol (2011) 9:604–15. doi: 10.1038/nrmicro2608 21747391

[8] Gatto M de Abreu MM Tasca KI Simão JC Fortaleza CM Pereira PC. Biochemical and Nutritional Evaluation of Patients With Visceral Leishmaniasis Before and After Treatment With Leishmanicidal Drugs. Rev da Sociedade Bras Medicina Trop (2013) 46(6):735–40. doi: 10.1590/0037-8682-0198-2013 24474015

[9] Ives A Ronet C Prevel F Ruzzante G Fuertes-Marraco S Schutz F. Leishmania RNA Virus Controls the Severity of Mucocutaneous Leishmaniasis. Science (New York NY) (2011) 331(6018):775–8. doi: 10.1126/science.1199326 PMC3253482 21311023

[10] Ribeiro-Gomes FL Sacks D. The Influence of Early Neutrophil-Leishmania Interactions on the Host Immune Response to Infection. Front Cell Infection Microbiol (2012) 2:59. doi: 10.3389/fcimb.2012.00059 PMC3417510 22919650

[11] Rosales C. Neutrophil: A Cell With Many Roles in Inflammation or Several Cell Types? Front Physiol (2018) 9:113. doi: 10.3389/fphys.2018.00113 29515456 PMC5826082

[12] Moll H. The Role of Dendritic Cells at the Early Stages of Leishmania Infection. Adv Exp Med Biol (2000) 479:163–73. doi: 10.1007/0-306-46831-X_14 10897418

[13] Favali C Tavares N Clarêncio J Barral A Barral-Netto M Brodskyn C. Leishmania Amazonensis Infection Impairs Differentiation and Function of Human Dendritic Cells. J Leukocyte Biol (2007) 82(6):1401–6. doi: 10.1189/jlb.0307187 17890507

[14] Contreras I Estrada JA Guak H Martel C Borjian A Ralph B. Impact of Leishmania Mexicana Infection on Dendritic Cell Signaling and Functions. PLoS Neglected Trop Dis (2014) 8(9):e3202. doi: 10.1371/journal.pntd.0003202 PMC4177750 25255446

[15] Jebbari H Stagg AJ Davidson RN Knight SC. Leishmania Major Promastigotes Inhibit Dendritic Cell Motility *In Vitro* . Infection Immun (2002) 70(2):1023–6. doi: 10.1128/IAI.70.2.1023-1026.2002 PMC127657 11796645

[16] Théry C Amigorena S. The Cell Biology of Antigen Presentation in Dendritic Cells. Curr Opin Immunol (2001) 13(1):45–51. doi: 10.1016/s0952-7915(00)00180-1 11154916

[17] Pinheiro NF Hermida MD Macedo MP Mengel J Bafica A dos-Santos WL. Leishmania Infection Impairs Beta 1-Integrin Function and Chemokine Receptor Expression in Mononuclear Phagocytes. Infection Immun (2006) 74(7):3912–21. doi: 10.1128/IAI.02103-05. C.OMMAJ.R.X.X.X. PMC1489695 16790764

[18] Wickham H. (2016). Springer-Verlag New York. Available at: https://ggplot2.tidyverse.org.

[19] Ludwig IS Geijtenbeek TB van Kooyk Y. Two Way Communication Between Neutrophils and Dendritic Cells. Curr Opin Pharmacol (2006) 6(4):408–13. doi: 10.1016/j.coph.2006.03.009 16750420

[20] Holbrook J Lara-Reyna S Jarosz-Griffiths H McDermott M. Tumour Necrosis Factor Signalling in Health and Disease. F1000Res (2019) 8:F1000 Faculty Rev–111. doi: 10.12688/f1000research.17023.1 PMC6352924 30755793

[21] Yin X Chen S Eisenbarth SC. Dendritic Cell Regulation of T Helper Cells. Annu Rev Immunol (2021) 39:759–90. doi: 10.1146/annurev-immunol-101819-025146 33710920

[22] Tibúrcio R Nunes S Nunes I Rosa Ampuero M Silva IB Lima R. Molecular Aspects of Dendritic Cell Activation in Leishmaniasis: An Immunobiological View. Front Immunol (2019) 10:227. doi: 10.3389/fimmu.2019.00227 30873156 PMC6401646

[23] Nicolás-Ávila JÁ. Adrover JM Hidalgo A. Neutrophils in Homeostasis, Immunity, and Cancer. Immunity (2017) 46(1):15–28. doi: 10.1016/j.immuni.2016.12.012 28099862

[24] Afonso L Borges VM Cruz H Ribeiro-Gomes FL DosReis GA Dutra AN. Interactions With Apoptotic But Not With Necrotic Neutrophils Increase Parasite Burden in Human Macrophages Infected With *Leishmania Amazonensis* . J Leukocyte Biol (2008) 84:389–96. doi: 10.1189/jlb.0108018 18483206

[25] Tavares N Afonso L Suarez M Ampuero M Prates DB Araújo-Santos T. Degranulating Neutrophils Promote Leukotriene B4 Production by Infected Macrophages To Kill Leishmania Amazonensis Parasites. J Immunol (2016) 196(4):1865–73. doi: 10.4049/jimmunol.1502224 26800873

[26] Geijtenbeek TB den Dunnen J Gringhuis SI. Pathogen Recognition by DC-SIGN Shapes Adaptive Immunity. Future Microbiol (2009) 4(7):879–90. doi: 10.2217/fmb.09.51 19722841

[27] Koppel EA Saeland E de Cooker DJ van Kooyk Y Geijtenbeek TB. DC-SIGN Specifically Recognizes Streptococcus Pneumoniae Serotypes 3 and 14. Immunobiology (2005) 210(2-4):203–10. doi: 10.1016/j.imbio.2005.05.014 16164027

[28] Ceccaldi PE Delebecque F Prevost MC Moris A Abastado JP Gessain A. DC-SIGN Facilitates Fusion of Dendritic Cells With Human T-Cell Leukemia Virus Type 1-Infected Cells. J Virol (2006) 80(10):4771–80. doi: 10.1128/JVI.80.10.4771-4780.2006 PMC1472089 16641270

[29] Geijtenbeek TB Kwon DS Torensma R van Vliet SJ van Duijnhoven GC Middel J. DC-SIGN, a Dendritic Cell-Specific HIV-1-Binding Protein That Enhances Trans-Infection of T Cells. Cell (2000) 100(5):587–97. doi: 10.1016/s0092-8674(00)80694-7 10721995

[30] Thépaut M Luczkowiak J Vivès C Labiod N Bally I Lasala F. Dc/L-SIGN Recognition of Spike Glycoprotein Promotes SARS-CoV-2 Trans-Infection and can be Inhibited by a Glycomimetic Antagonist. PLoS Pathog (2021) 17(5):e1009576. doi: 10.1371/journal.ppat.1009576 34015061 PMC8136665

[31] Blanco P Palucka AK Pascual V Banchereau J. Dendritic Cells and Cytokines in Human Inflammatory and Autoimmune Diseases. Cytokine Growth Factor Rev (2008) 19(1):41–52. doi: 10.1016/j.cytogfr.2007.10.004 18258476 PMC2413068

[32] van Gisbergen KP Sanchez-Hernandez M Geijtenbeek TB van Kooyk Y. Neutrophils Mediate Immune Modulation of Dendritic Cells Through Glycosylation-Dependent Interactions Between Mac-1 and DC-SIGN. J Exp Med (2005) 201(8):1281–92. doi: 10.1084/jem.20041276 PMC2213143 15837813

[33] Allenbach C Zufferey C Perez C Launois P Mueller C Tacchini-Cottier F. Macrophages Induce Neutrophil Apoptosis Through Membrane TNF, a Process Amplified by Leishmania Major. J Immunol (2006) 176(11):6656–64. doi: 10.4049/jimmunol.176.11.6656 16709824

[34] Hovius JW de Jong MA den Dunnen J Litjens M Fikrig E van der Poll T. Salp15 Binding to DC-SIGN Inhibits Cytokine Expression by Impairing Both Nucleosome Remodeling and mRNA Stabilization. PLoS Pathog (2008) 4(2):e31. doi: 10.1371/journal.ppat.0040031 18282094 PMC2242833

[35] Carneiro PP Conceição J Macedo M Magalhães V Carvalho EM Bacellar O. The Role of Nitric Oxide and Reactive Oxygen Species in the Killing of Leishmania Braziliensis by Monocytes From Patients With Cutaneous Leishmaniasis. PLoS One (2016) 11(2):e0148084. doi: 10.1371/journal.pone.0148084 26840253 PMC4739692

[36] Roma EH Macedo JP Goes GR Gonçalves JL Castro Wd Cisalpino D. Impact of Reactive Oxygen Species (ROS) on the Control of Parasite Loads and Inflammation in Leishmania Amazonensis Infection. Parasit Vectors (2016) 9:193. doi: 10.1186/s13071-016-1472-y 27056545 PMC4825088

[37] Figueiredo AB Serafim TD Marques-da-Silva EA Meyer-Fernandes JR Afonso LC. Leishmania Amazonensis Impairs DC Function by Inhibiting CD40 Expression *via* A2B Adenosine Receptor Activation. Eur J Immunol (2012) 42(5):1203–15. doi: 10.1002/eji.201141926 22311598

