# Supplementary figures and images for: DC-SIGN Mediates the Interaction Between Neutrophils and Leishmania amazonensis-Infected Dendritic Cells to Promote DC Maturation and Parasite Elimination
Source: Front Immunol. 2021 Nov 1;12:750648. doi: 10.3389/fimmu.2021.750648 (PMC8591281; doi:10.3389/fimmu.2021.750648)

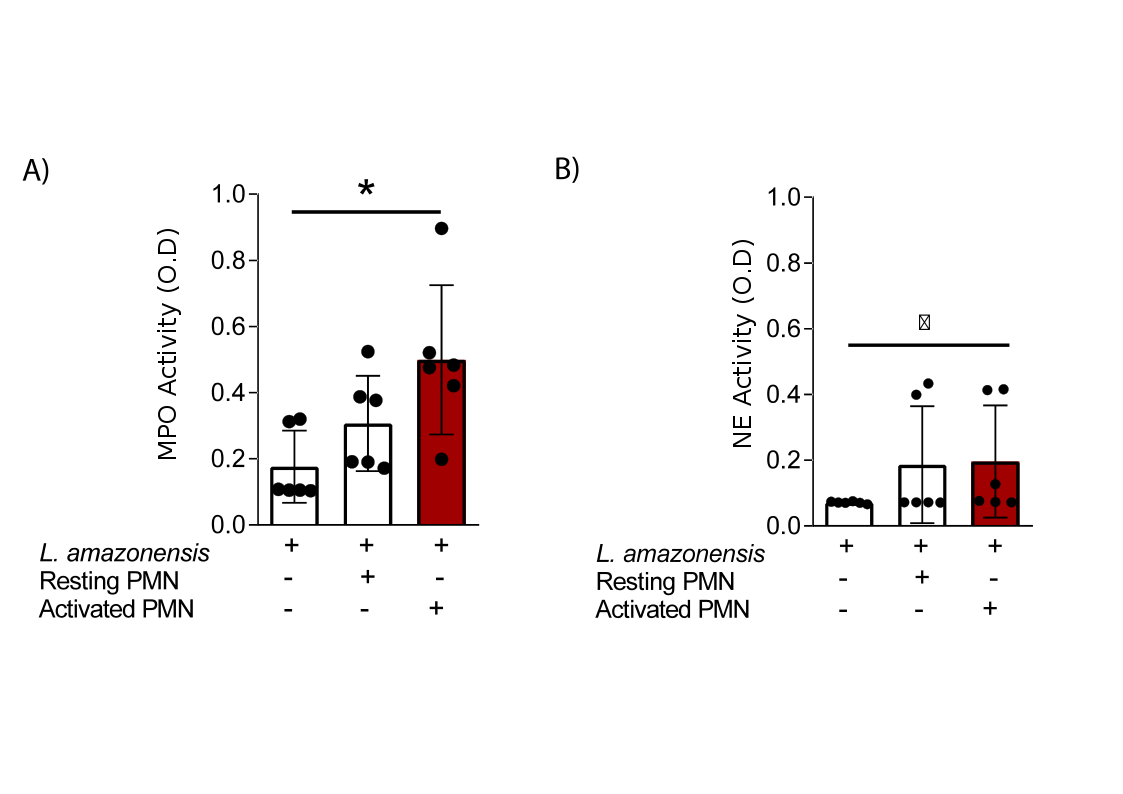

Supplement: Supplementary Figure 1 — Assessment of Neutrophil enzymes activity in PMN-infected DCs cocultures. The enzymatic activity of MPO (A) and Neutrophil elastase (B) were measured in coculture supernantants. Kruskal-Wallis test with Dunn post-test. *p < 0,05. [file Image_1.tiff]

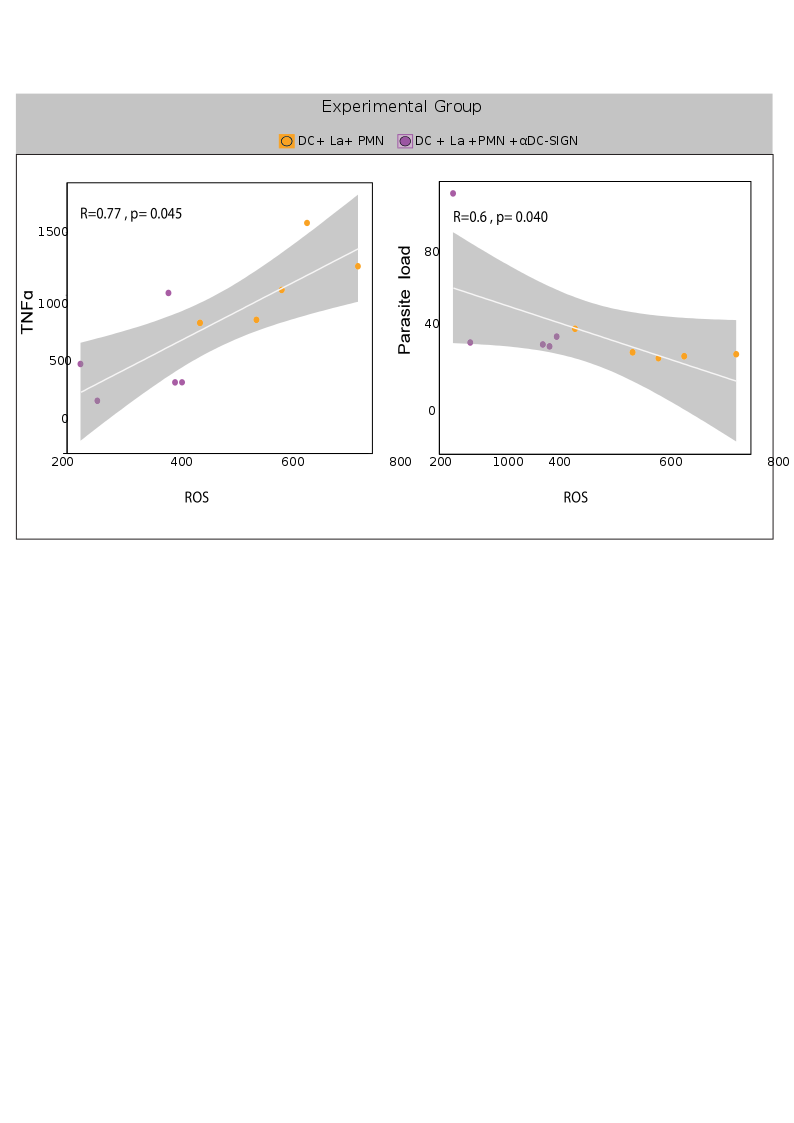

Supplement: Supplementary Figure 2 — Spearman correlations between TNFα and ROS production. Briefly, monocyte-derived DCs were cultured with IL-4 and GM-CSF for 7 days. At the end of this period, fully-differentiated DCs were infected with metacyclic L. amazonensis promastigotes in the proportion of 10 parasites per cell. Subsequently, DCs were centrifuged to remove non-internalized parasite and incubated with fibronectin-activated neutrophils for 12 hours. Supernatant was collected for assessment of TNFα production. ROS production was determined by flow cytometry. *p < 0,05. Correlations were tested by a two-tailed non-parametric Spearman rank test. [file Image_2.tiff]

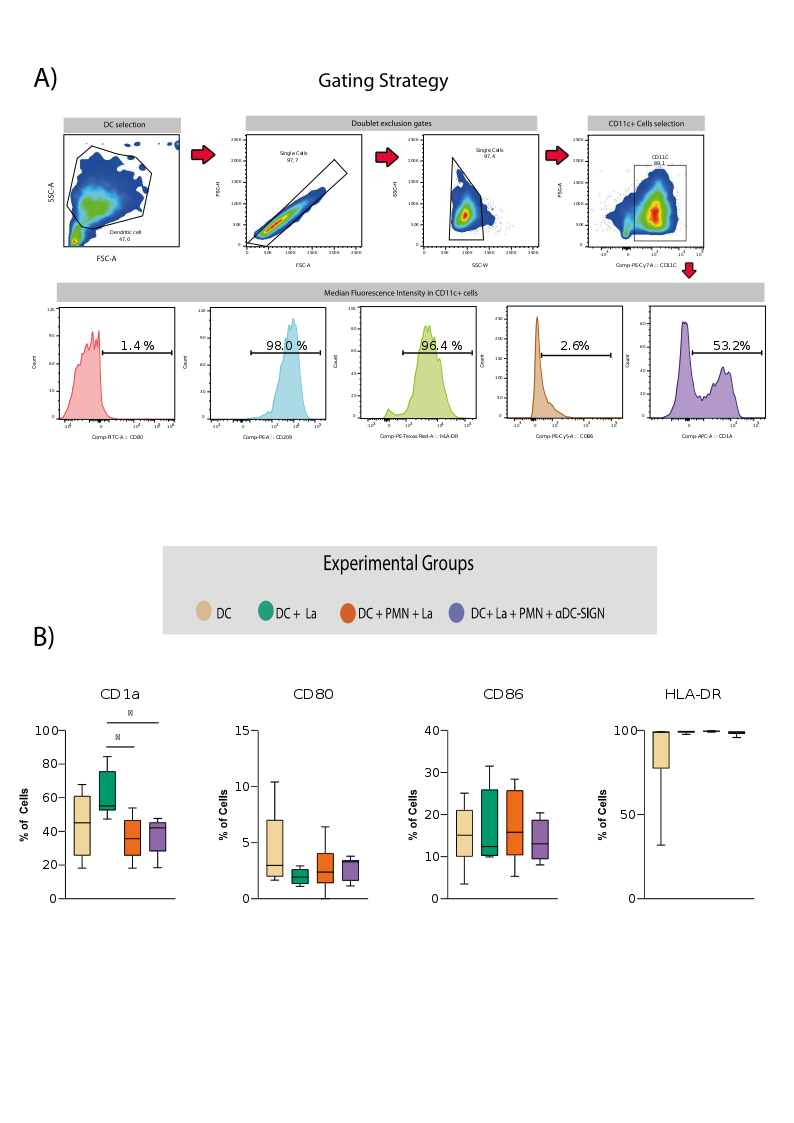

Supplement: Supplementary Figure 3 — Flow cytometry analysis of maturation -related molecules in DCs. (A) Gating strategy for DCs identification and (B) frequency of CD1a, CD80, CD86, and HLA-DR expression. Kruskal-Wallis test with Dunn post-test. *p < 0,05. [file Image_3.tiff]

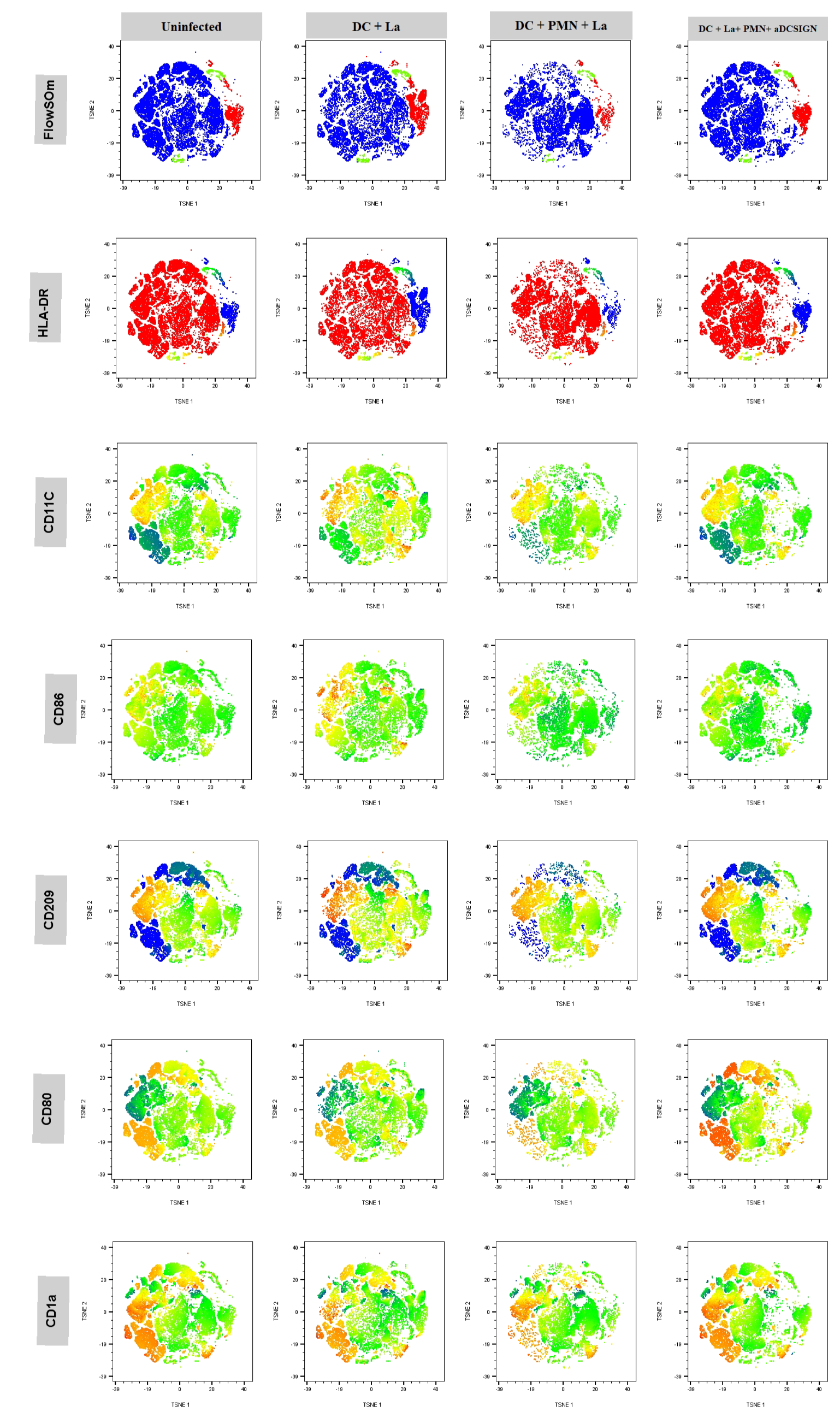

Supplement: Supplementary Figure 4 — High dimensional flow cytometry characterization of DC subpopulations. (A) t-distribute stochastic neighbor embedding (tSNE) and FlowSOM clusterization considering the expression of HLA-DR,CD80, CD86, CD1a, CD16, and CD209 across experimental conditions. [file Image_4.tif]
