# Supplementary material for: DC-SIGN Mediates the Interaction Between Neutrophils and Leishmania amazonensis-Infected Dendritic Cells to Promote DC Maturation and Parasite Elimination
Source: Front Immunol. 2021 Nov 1;12:750648. doi: 10.3389/fimmu.2021.750648 (PMC8591281; doi:10.3389/fimmu.2021.750648)
Supplement: Supplementary file 5 [file DataSheet_1.pdf]

Supplementary Table I. List of antibodies used for flow cytometry assays

Antibody	Fluorochrome	Supplier	Clone	Type	Reference
**CD1a**	APC	BD Bioscience	HI149	Primary	560945
**CD11c**	Pe-cy7	BD Bioscience	B-ly6	Primary	561356
**CD80**	FITC	BD Bioscience	L307.4	Primary	555683
**CD86**	Pe-Cy5	BD Bioscience	Clone IT2.2 (RUO)	Primary	555666
**CD209**	Pe	BD Bioscience	DCN46	Primary	551265
**HLA-DR**	BV711	Biolegend	L243	Primary	307602
